# Medical informatics and digital health multilingual ontology (MIMO): A tool to improve international collaborations

**DOI:** 10.1016/j.ijmedinf.2022.104860

**Published:** 2022-11

**Authors:** Arriel Benis, Julien Grosjean, Kévin Billey, Gustavo Montanha, Verena Dornauer, Mihaela Crișan-Vida, Werner O Hackl, Lăcrămioara Stoicu-Tivadar, Stéfan J. Darmoni

**Affiliations:** a Faculty of Industrial Engineering and Technology Management, Holon Institute of Technology, Israel; b Faculty of Digital Technologies in Medicine, Holon Institute of Technology, Israel; c Department of Biomedical Informatics, Rouen University Hospital, France; d LIMICS Laboratory of Medical Informatics and Knowledge Engineering in e-Health, INSERM U1142, Sorbonne Université, France; e Centro Hospitalar de Entre o Douro e Vouga EPE: Santa Maria da Feira, Aveiro, Portugal; f Institute of Medical Informatics, Private University for Health Sciences, Medical Informatics and Technology, Hall in Tirol, Austria; g Department of Automation and Applied Informatics, University Politehnica Timișoara, Timișoara, Romania; h European Federation for Medical Informatics, Le Mont-sur-Lausanne, Switzerland

**Keywords:** Vocabulary, Controlled, Communication, Information Services, Language, Digital Health, Translations, Biomedical Ontologies, Intersectoral Collaboration, API, Application Programming Interface, DSM, Diagnostic and Statistical Manual of Mental Disorders, EFMI, European Federation for Medical Informatics, EU, European Union, HeTOP, Health Terminology/Ontology Portal, HIIC, Health Informatics for Inter-regional Cooperation, HosmartAI, HOspital Smart development based on Artificial Intelligence, ICD, International Classification of Diseases, ISO, International Organization for Standardization, MedDRA, Medical Dictionary of Regulatory Activities, MeSH, Medical Subject Headings, MIE, Medical Informatics Europe Conference, MIMO, Medical Informatics and Digital Health Multilingual Ontology, NCIt, National Cancer Institute Thesaurus, OMT, Online Machine Translation, OWL, Web Ontology Language, SNOMED CT, Systematized Nomenclature of Medicine - Clinical Terms, STC, Special Topic Conference, UMLS, Unified Medical Language System, WG, Working Group

## Abstract

**Background:**

Even if English is the leading language for international communication, it is essential to keep in mind that research runs at the local level by local teams generally communicating in their local/national language, especially in Europe among European projects.

**Objective:**

Therefore, the European Federation for Medical Informatics - Working Group on Health Informatics for Inter-regional Cooperation” has one objective: To develop a multilingual ontology focusing on Health Informatics and Digital Health as a collaboration tool that improves international and, in particular, European collaborations.

**Results:**

We have developed the Medical Informatics and Digital Health Multilingual Ontology (MIMO). Hosted on the Health Terminology/Ontology Portal (HeTOP), MIMO contains around 1,000 concepts, 460 MeSH Descriptors, 220 MeSH Concepts, and more than 300 newly created concepts. MIMO is continuously updated to comprise as recent as possible concepts and their translations in more than 30 languages. Moreover, the MIMO’s development team constantly improves MIMO content and supporting information. Thus, during workshop discussions and one-on-one exchanges, the MIMO team has collected domain experts’ opinions about the community’s interests and suggestions for future enhancements. Moreover, MIMO will be integrated to support the annotation and categorization of research products into the HosmartAI European project involving more than 20 countries around Europe and worldwide.

**Conclusion:**

MIMO is hosted by HeTOP (Health Terminology/Ontology Portal), which integrates 100 terminologies and ontologies in 55 languages. MIMO is freely available online. MIMO is portable to other knowledge platforms as part of MIMO’s main aims to facilitate communication between medical librarians, translators, and researchers as well as to support students’ self-learning.

## 1 Introduction

### 1.1 Background

Scientific projects, particularly in the Health Sciences and Technologies field, are nowadays increasingly multi-centric by involving partners with different capabilities, skills, and expertise from different countries [1], [2]. This complexity always happens in European projects involving other partners using at least one European language, even if English became the scientific “lingua franca” used for international communication. Indeed, English is massively used compared to other languages for scientific publications. This is not due to the English fluency of the authors owing to different research evaluation processes highlighting the importance of publishing in English-speaking international journals [3]. However, it is essential to keep in mind that research runs at the local level by local teams generally communicating in their own local or national language [4], [5].

Digital Health and Health Informatics are at the crossroads of medicine and health sciences, computer science and engineering, information and communication sciences, mathematics, statistics, technology, and innovation management. However, Digital Health focuses on using information and communication technologies to deliver healthcare services to promote better health and well-being. Health informatics deals broadly with data, information, knowledge, and decision-making for healthcare professionals and organizations. In both fields, the interactions between different kinds of actors, such as healthcare informaticists, practitioners, managers, and customers and their relatives, are critical and complex. However, the main objective of Digital Health is to support the delivery of healthcare in a safe, efficient, and accessible way to and for everyone.

The European Federation for Medical Informatics (EFMI) is built around 30 member-countries affiliated societies [6]. One of the objectives of EFMI is to encourage academic and professional collaboration and cooperation between all its members (both from academia and industry), to enhance health and medical informatics research, education, knowledge, and industrial transfer. Thus, the EFMI Working Group (WG) on Health Informatics for Inter-regional Cooperation (HIIC) [7] deals with enhancing the quality of the exchanges between international health informatics research partners.

### 1.2 Aims and objectives

In the 1990s, some glossaries, thesauri, and domain models, comprising a few hundred to 2,000, were developed in Medical Informatics. Two teams led these projects, one from Missouri, USA [8], [9] and the other from Liverpool, UK [10]. These paper-printed vocabularies were in English only. Moreover, according to our knowledge, there is no similar prior work dealing with multilingual issues. One of the EFMI WG HIIC aims to develop a frequently updated multilingual controlled vocabulary focusing on Health Informatics and Digital Health as a collaboration tool that improves international and, more particularly, European cooperation [11]. Thus, the leading question of this research relates to the definition of a methodology allowing the continuous development and improvement of the multilingual ontology of medical informatics.

In this manuscript, our objective is to present the development process of the multilingual ontology on health informatics (and beyond, digital health, including health information sciences), called MIMO (Medical Informatics Multilingual Ontology). The MIMO project mainly focuses on European languages within the umbrella of EFMI. However, it includes other languages worldwide to be accessible to the largest possible part of the scientific community. Moreover, we show that MIMO is favorably welcomed by the “Health Informatics and Digital Health” community both at the individual and collective levels.

## 2 Material and methods

The development, management, and continuous improvement of MIMO are challenging. MIMO was built in five steps. The first version of MIMO was developed in 2018 as a flat dictionary [12]. Said version was a proof-of-concept based on the first three steps of the MIMO development methodology reported below. Then the current version of MIMO consisted of combining knowledge extraction (from existing controlled vocabularies), and knowledge generation (by getting new terms from domain experts, and from native speakers of the different included languages in MIMO, translations of previously translated ones).

The first step of the MIMO project consisted in defining the list of the official languages of the EFMI member countries.

In 2021, the 27 member countries of the European Union (EU) had a population of 517 million inhabitants and 746 million, including the people of geographical Europe and associated countries. Currently, there are 24 official languages in the EU: Bulgarian, Croatian, Czech, Danish, Dutch, English, Estonian, Finnish, French, German, Greek, Hungarian, Irish, Italian, Latvian, Lithuanian, Maltese, Polish, Portuguese, Romanian, Slovak, Slovene, Spanish and Swedish, and 36 languages in Europe (e.g., Russian) [13], [14]. According to their origin and similarities, all these languages can be classified into eight language groups [15].

The first version of MIMO was a flat dictionary as the third step with some initial main categories of terms such as “Computer (Hardware),” “Computer (Software),” “Medical device,” “System,” “Programming,” “Organization,” “Journal,” and “Application field.” An initial set of 160 terms populated the dictionary, and when relevant related acronyms existed for some terms, they were also added. For example, “International Conference on Informatics, Management, and Technology in Healthcare” and “ICIMTH,” in English, French, Romanian, and Hebrew, the main languages known as a native-speaker level by the EFMI dedicated team to this project.

The second step consisted in associating each of the 36 languages that have been previously identified with the relevant ISO 639 code to facilitate the subsequent implementation steps [16].

Then, as a third step, a simple automated translation process, using an online machine translation (OMT) tool supporting over 100 languages at various levels, has been implemented. We listed the terms and used an OMT [17] function for each of the 36 targeted languages. The OMT service was able to detect the language of the submitted term with high accuracy and then translate it with variable correctness to target languages according to the ISO 639 codes previously selected. As a result of this automated translation process, a flat file has been generated. However, as a part of the quality control point of this step, the MIMO team has pointed out the lack of translated accuracy for at least half of the 36 languages. This lack of accuracy was noticed by having, for example, English terms used in the translation to another language instead of said language itself or for languages known by the team inaccuracy (e.g., informatics translate in Hebrew as “Library Science” instead of “Information Science”). This issue is due to the different levels of maturity of the translation capabilities of the service. These results are consistent with other studies focusing on other fields [18] and specifically in health [19]. Accordingly, human language speakers are involved in validating, improving, and expanding the dictionary over time.

The fourth step to create MIMO was the creation of a thesaurus to be used to build an ontology. The MIMO team has analyzed the main controlled vocabularies in health and being a part of UMLS [20]: MeSH [21], SNOMED CT [22], NCIT [23], MedDRA [24] or ICD (in their ninth, 10th, and 11th revision) [25]. Based on this manual review by two medical informaticians (AB & SJD), the MeSH thesaurus was assessed as having the most comprehensive number of medical informatics concepts. Indeed, the MeSH thesaurus is the unique reference terminology, where a tree describing medical informatics and, more broadly, information science exists. Accordingly, to complete this thesaurus as exhaustively as possible, the MIMO team has decided to expand the concepts to be included not only extracted from the MeSH hierarchy “medical informatics” but to be extracted from the broader concept “information science.” In other words, there is an “IS_A” relation between “medical informatics” and “information science,” where “medical informatics” is the narrower concept. No such tree exists in SNOMED CT [22] or in NCIT [23], where only a tree of “software” exists in these two reference terminologies, and both are currently integrated into MIMO.

The MeSH thesaurus has five different types of concepts: MeSH Descriptors, MeSH Supplementary Concepts, MeSH Concepts, MeSH Resource Types, and MeSH qualifiers. Two of which were used to build MIMO: MeSH Descriptors and MeSH Concepts of the current 2021 version. Terms in a MeSH record, which are strictly synonymous with each other, are grouped in a category called a “MeSH Concept.” Each MeSH record consists of one or more concepts, and each concept consists of one or more synonymous terms. Each MeSH concept is related to a MeSH Descriptor. For example, the MeSH Descriptor “Diagnostic and statistical manual of mental disorders” has four related MeSH Concepts: DSM-II, DSM-III, DSM-IV, and DSM-V [26]. These four MeSH concepts were integrated into MIMO. Four relations exist between MeSH Descriptors and MeSH Concepts [21]:

1. Preferred Term (PT), the MeSH Descriptor, and the MeSH Concept are accurate synonyms;
2. Broader Term (BT), the MeSH Concept is hierarchically more general than the MeSH Descriptor;
3. Narrower Term (NT), the MeSH Concept is hierarchically more limited than the MeSH Descriptor;
4. Related Term (RT), the MeSH Concept, and the MeSH Descriptor are related, is a “see also” relation, without any hierarchical relation (e.g., prison RT prisoners).

Thus, in the example of the MeSH Descriptor “Diagnostic and statistical manual of mental disorders,” MIMO has applied the relations between this MeSH Descriptor and the four MeSH Concepts and were integrated into MIMO, which is not the case in the MeSH thesaurus.

The previous concepts included in the dictionary were disseminated in the various hierarchies of MIMO. Specific hierarchies were created with medical concepts, which do not exist in other systems, methods, and terminologies of reference: in particular, “Supervisory Control And Data Acquisition (SCADA)” [27], [28] or “diseases linked to digital health” [29], [30]. Some added concepts were coming from SNOMED CT, MedDRA, and NCIT.

The structure of the tree-based MeSH has implications for the multilingual ontology by pointing out the exact or near translation of terms, such as native speakers provide them (e.g., humans or knowledge-sharing platforms such as Wikipedia). Nowadays, MIMO is built as an ontology according to the different relationships and dependencies defined in the hierarchy of terms. For example, translations are defined as follows: is_a_human_translation, is_a_terminology-based_translation.

In the fifth step, because disseminating the information and the knowledge included in MIMO is a critical phase, the MIMO team had to define which kind of platform would be relevant to:

• Host and maintain it efficiently over time,
• Support integration with other existing terminologies, classifications, and ontologies in health and engineering sciences,
• Provide the end-user with a user-friendly interface that will encourage using the dictionary as a day-to-day tool.

The MIMO team’s interest focused on the following terminology server: a cross-lingual health terminology and ontology server (HeTOP; URL: https://www.hetop.eu/hetop/rep/en/terminologies/ [31]), which contains more than 2 million concepts, 100 terminologies, and ontologies, 55 languages, including non-European languages (e.g., Mandarin, Arabic, Korean or Japanese) [32]. Moreover, the Generic Model of HeTOP is compatible with the ISO 25,964 standard and allows interoperability with other vocabularies platforms [33], [34], [35]. The proof-of-concept of the MIMO was integrated, in mid-2019, into the HeTOP cross-lingual terminology server and is available to the consortium using an identification [36]. Furthermore, we have evaluated the interest of MIMO by the “Health Informatics and Digital Health” community both at the individual and collective levels.

## 3 Results

### 3.1 MIMO as a community freely available tool

MIMO is currently available as a component integrated into the HeTOP server(URL: https://www.hetop.eu/hetop/rep/en/EFMIMIMO/). Among the 100 terminologies and ontologies integrated into HeTOP, only 18 are also integrated into the UMLS [37]. Termino-ontologies are regularly updated and are accessible via a website and a dedicated web service. HeTOP has been designed as a reference multi-terminology and cross-lingual portal to help librarians, translators, students, and medical professionals retrieve resources and knowledge across various complex medical fields.

On 27 April 2022, MIMO contained 1,261 concepts, 666 from MeSH, 136 from NCIT, 177 from SNOMED CT, and 282 newly created concepts. We plan to integrate around 2,500 MIMO concepts at the end of the HosmarAI project in 2024 and continue to expand it in the future.

The advantages of using HeTOP as the hosting platform of MIMO are:

• The navigability capabilities between the different available thesauruses and ontologies allowing discovering if a search term exists in MIMO and provided resources;
• The interface is translated into 12 languages (partially or fully processed both by domain experts and online AI-based systems) to facilitate the platform used by a large community not always comfortable with English, similarly to other well-known environments;
• The possibility to get dynamically generated links to relevant search query results on PubMed, or at this time, CISMeF, the Catalog and Index of French Language Health Resources on the Internet [38];
• The frequent updates of the terminologies and ontologies hosted allowing to be enriched thanks to alignment processes MIMO, when relevant dynamically;
• HeTOP comprises capabilities of controlled vocabularies alignment and mapping [39], [40]. Thus, the initial terms included in MIMO have been automatically aligned with the same ones existing in other terminologies and ontologies existing in HeTOP. Then the MIMO terms were mapped in the vocabularies wherein its terms were discovered. It allowed expanding MIMO automatically. Nevertheless, health informatics and digital health fields are evolving quickly. Some terms included in MIMO are not discovered in other HeTOP vocabularies. This is the most advanced added value of this research (i.e., having an up-to-date vocabulary comprising terms related to cutting-edge research and technologies). The alignment and mapping processes are periodically run to ensure the availability of the most enriched version of MIMO;
• The possibility of involving a native language speaker and domain expert in the translation process or validation to a specific language without an open system.

**Fig. 1:**
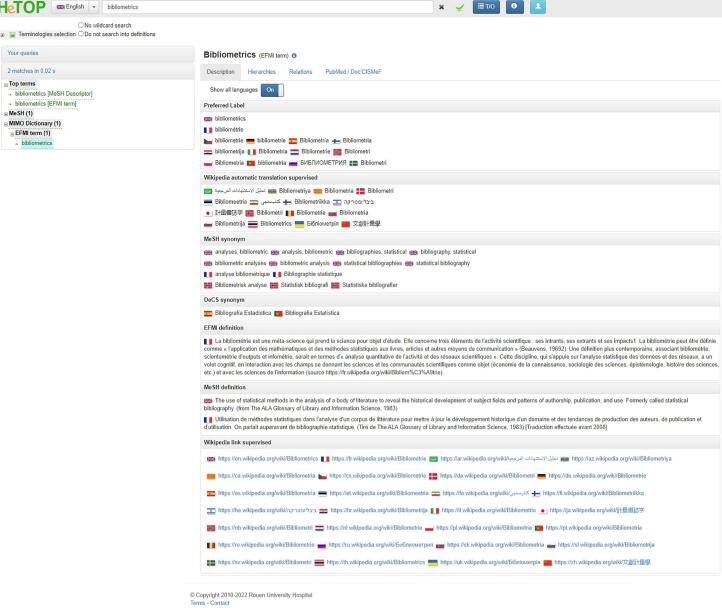
Example of HeTOP response for a query with the term “bibliometrics”.

Fig. 1 shows an example of a HeTOP response for a query with the term “bibliometrics.” Using the interface in its English version (the user can switch to a different language by using the dropdown list appearing on the top-left side of the screen) and asking for results from all the available terminologies and ontologies (by selecting in the tree of the available terminologies “Terminologies selection”) allowing a matrix navigation (among languages and terminologies). The following description sections are available:

• Preferred Label: the MeSH Descriptor and its translated versions as provided by native speaker level domain experts; herein, e.g., “bibliometrics” is translated into 13 languages;
• We have developed a specific generic algorithm (currently named WikiMeSH) to propose an extended translation of any thesaurus based on Wikipedia pages [41]. A mapping was realized between each MeSH descriptor (preferred terms and synonyms) and corresponding Wikipedia pages by using the Wikipedia API (Application Programming Interface) [42]. Among the top 20 languages of this study, seven currently have no MeSH translations: Arabic, Catalan, Farsi (Iran), Mandarin Chinese, Korean, Serbian, and Ukrainian. For these seven languages, WikiMeSH is proposing translations, for example, of 47 % to Arabic and 34 % to Serbian [43] ;
• MeSH synonyms: Synonyms and equivalent terms and concepts according to the MeSH but less used or less popular or more generally “related terms”; herein, we got “bibliometrics” MeSH synonyms in British English, American English, and Danish;
• DeCS synonyms: based on MeSH, DeCS (in Spanish: Descriptores en Ciencias de la Salud, in English: Health Sciences Descriptors) is a controlled and structured vocabulary in four languages (Spanish, Portuguese, French, and English). It expands the MeSH thesaurus, containing over 5,000 health concepts. The DeCS project started during the 1970 s [44]; herein, we got “bibliometrics” translated by domain experts to Spanish and Portuguese;
• Définition CISMeF: definition of the preferred label, in French, when available, manually translated by CISMeF librarians and health professionals [38];
• MeSH definition: definition of the preferred label, in English, extracted from the MeSH [21];
• Wikipedia link: Links to the relevant and available Wikipedia pages in the different languages ran into MIMO to provide the users with more definitions and information as the Wikipedia community writes and shares it. It is critical to point out that Wikipedia’s community controls the content of Wikipedia, but this does not ensure that it is validated by medical informatics and digital health vocabularies experts.

The HeTOP user can search for a term only in MIMO. To do this, the user must select only MIMO in the list “Terminologies selection”.

### 3.2 Health informatics and digital Health” community interest in MIMO

As a part of the development of MIMO and keeping in mind the need for its continuous improvement, getting feedback is essential, both at the individual and collective level. We received inputs broadly (1) during workshop discussions and one-on-one exchanges and (2) in integrating MIMO into the HosmartAI European project for supporting multilingual knowledge and research products dissemination.

### 3.3 Medical informatics community feedback on the MIMO concept

Before starting the MIMO project in its current format, the EFMI WG HIIC had organized, during scientific meetings, a few workshops and panels focusing on cooperation and collaboration in the health community during international scientific meetings. During these events, we have discussed with the audience the relevancy and our research on a dedicated multilingual controlled vocabulary (ICIMTH 2019 [12]; EFMI and the Romanian Society of Medical Informatics - Adapting Education during COVID-19 Pandemic (online event) [45]; MIE 2021 [46]; STC 2021/HosmartAI Satellite event [47]). We got encouraging comments on the MIMO concept from the health informatics and digital health community. Indeed, end-users shared an interest in integrating and translating knowledge for the clinical practice and health services customers’ benefits. Moreover, from a technological perspective, one frequent feedback was that a multilingual ontology focusing on medical informatics and digital health, such as MIMO, is fundamental to the future of health information technology systems. It must support the perpetual inclusion of new technological tools in the health-related process (i.e., diagnosis, treatment, monitoring, and follow-up). Moreover, one crucial community comment is that MIMO should be open-sourced like other knowledge management tools to allow its dissemination, growth, and maintenance. Therefore, MIMO is freely accessible on HeTOP to the entire scientific community and on-demand as an OWL (Web Ontology Language) file and via the HeTOP API.

Moreover, it was also important to have clinicians’ feedback about potential applications to daily practice. On the one hand, we got these non-formal, verbal, and unstructured inputs, from members of the MIMO team (GM, VD), and on the other hand, from a few participants at the dedicated meetings reported above. A large part of them are looking at MIMO as the pillar of tools that allows them to find semantic relations in their clinical notes and their knowledge base (e.g., scientific papers, reports) to be able to support and justify some digital health-oriented advice given to patients (e.g., using a smartwatch for monitoring physical activity, preferring a non-invasive, robot-assisted surgery) as a part of an evidence-based medicine approach.

### 3.4 Integration with the “HOspital SMART development based on artificial Intelligence” European project

Since January 2021, MIMO development has been partially granted by the Horizon 2020 European project “HOspital SMART development based on Artificial Intelligence” (AI; HosmartAI) [48]. HosmartAI is a European project involving 24 partners in 12 countries. HosmartAI promotes an effective and efficient healthcare system transformation through technological developments in AI and robotics. HosmartAI is creating a common open integration platform, called HosmartAI Marketplace, with tools to facilitate and measure the benefits of integrating digital technologies (such as robotics and AI) in the healthcare system. The main objective herein is to improve the interoperability among the various tools of this project. Accordingly, MIMO has been selected for allowing the description with the MIMO concepts of the different products available in the HosmartAI Marketplace. The main objectives of this integration are to facilitate (1) the uses of the research products developed by the 24 partners in 12 countries, and (2) the daily communication practice of the different teams in one of their mother tongues.

With this objective and the previous inclusion of the MeSH tree on “information science,” we noticed that its granularity is limited (e.g., the term “robotics” appears a few times in MeSH, but not the biomedical engineering terms related to the components of a robot). Therefore, this framework expands MIMO to provide a more exhaustive and up-to-date controlled vocabulary relevant to hospital informatics.

Moreover, MIMO can be integrated with and into other platforms. To this end, MIMO is accessible as an OWL file and a Web service via the HeTOP API for the HosmartAI consortium to be integrated into the platforms developed.

In the context of the HosmartAI project, to support the overall communication of different actors of the projects and the users of its products efficiently and effectively, overtime MIMO is:

• Expanding the number of concepts (from nearly-one thousand to a few thousand);
• Expanding the number of categories and sub-categories (to a few dozen). For example, robotics, Internet-of-Things, and Human-Computer Interactions, in the context of digital health and artificial intelligence in healthcare (i.e., hospitals) should be expanded;
• Involving native speakers of the different European languages for evaluating, validating, or correcting the automatically imported or translated concepts, as we did for Portuguese, Romanian, and soon Swedish.

## 4 Discussion

### 4.1 Overview

The development of multilingual controlled vocabularies such as thesauri and ontology related to the Medical Informatics and Digital Health fields for enhancing the quality and efficiency of international projects is challenging. The MIMO developers aim to support all the actors interacting in multilingual projects at all the levels of professional health (industry) communication (e.g., education, research, engineering, knowledge dissemination, marketing), such as the one required in the European context. The MIMO benefits from the computational, scientific, and linguistic resources offered by the freely accessible HeTOP environment.

### 4.2 Strengths and limitations

The globalization process of the healthcare system, looking at the clinical practitioners, administrative teams, technology developers, and enablers, is long and complex. Indeed, the rules and regulations are specific to each country. Moreover, the spoken language in each country is specific, so the same word can have another meaning when translated.

The main strength of MIMO is the capacity to provide to the different actors of a healthcare technologies-related project to communicate in a more unified language framework. MIMO gives the possibility to search in their native language a translation of medical informatics, or more broadly, a digital health-related term currently in another European language, and check that the meaning is the same for both in a third one, like English.

The second strength of MIMO is the capability to combine automated validated and domain-expert translations. For languages primarily practiced in Europe and around the World, the automated and validated translations from different sources cover a large part of the MIMO terms and concepts. Nevertheless, native language speakers’ domain experts’ involvement allows getting an up-to-date terminology and thesaurus in a range of different cases, such as:

1. New terms and concepts that are still not in existing terminologies and ontologies, such as MeSH,
2. For less popular languages with a meager rate or non-existing translations.

A third strength of MIMO is that HeTOP hosts it, and this allows:

1. Hosting and maintaining it efficiently over time;
2. Supporting the mapping with other existing terminologies, classifications, and ontologies in health and engineering sciences;
3. Providing to the end-user a user-friendly interface that will encourage using MIMO as a day-to-day tool; each MIMO concept has a dedicated URI that can be shared with end-users across the globe.

The main strength of MIMO is to be up-to-date with recent and new terms and concepts related to new technologies.

However, MIMO has some limitations. Indeed, for non-frequent languages, a native speaker level domain expert with multilingual capabilities is needed to translate from one language to another or validate automated translation (e.g., based on online services such as Google Translate, Bing Translator). Thus, for this domain expert’s time-consuming involvement, it is necessary to engage them in a community also giving translation peer-review, validation, and enhancing domain-related knowledge to expand the new terms’ translation capabilities.

Moreover, not all the terms and concepts of the MIMO are fully translated into all the supported languages simultaneously. This time-based discrepancy and human resources limitations are, like in other multilingual projects (i.e., Wikipedia [41], Wiktionary [49]), well-known issues that can be managed by building a community of volunteers worldwide.

Medical informatics and digital health are dynamic fields with frequent new inputs (i.e., new technologies, systems, methods, algorithms, new terms and concepts). Therefore, the MIMO core team and community must have up-to-date knowledge and define a flow for integrating new terms and concepts (e.g., which languages are the initial inputs and how are they curated? What is the frequency of the MIMO updates release). Furthermore, this is in continuation of the previous point.

### 4.3 Future perspectives.

The next scheduled steps of the MIMO consist in:

• Developing a community of volunteer domain experts in the different fields of medical informatics and digital health having multilingual capabilities for expanding both the translations of the existing terms and developing a cross-translators (humans and AI-based systems) validation process;
• Expanding MIMO to other non-European languages, such as Arabic, Chinese, and Russian, as part of the UN official languages in addition to English, French, and Spanish [50];
• Developing tools allowing to capture existing translations in open data resources and so expanding MIMO to other languages supported by multilingual collaborative communities [43];
• Adding vocabularies of trending or emerging fields in medical informatics and digital health, such as “One Digital Health” [51], “Accident and Emergency Informatics” [52], robotics in healthcare systems and industry [53];
• Moving MIMO to a complete multilingual ontology to facilitate its maintenance and expansion to support efficient international health technologies-related projects [54].

## 5 Conclusion

Developing persisting and fruitful inter-regional and international projects, cooperation, and collaboration is an endless challenge. Indeed, workspace virtualization, online-based communication, and the continuously increasing use of computational tools facilitate the search for new academic and industrial research partners. Nevertheless, communication misunderstandings can lead to delays and failure of potentially disruptive innovation projects [55], [56]. Therefore, managing complex projects in medical informatics and digital health fields requires a basic but proper understanding of different relevant vocabularies (e.g., technology, informatics, engineering, health, and medical sciences) [57], [58].

MIMO aims to provide a multilingual tool focusing specifically on health informatics and digital health technologies. The methodology empirically implemented for continuous development of MIMO strives to have this controlled vocabulary as a pillar component of an environment supporting health sciences information professionals’ different activities, facilitating the mutual understanding of researchers both from academia and industry, and enhancing students’ self-learning in a global world with mobility opportunities. It is essential to integrate existing terminological resources in a unified way (e.g., in UMLS, MeSH, NCIt, SNOMED CT) and to reduce translation-based misunderstanding by involving native language speakers directly (as a part of a working group) or indirectly (via the social platforms. Therefore, MIMO is a timely opportunity to develop and improve scientific, technical, educational, and industrial partnerships in medical informatics and digital health.

**Summary table**.

What was already known on the topic:

1. Developing long-term international cooperation is challenging in conducting research. Creating international partnerships looks more accessible than ever but can fail due to language differences and misunderstandings.
2. Managing complex projects in medical informatics and digital health fields requires a basic but proper understanding of the terminologies both from the technological and medical sides.

What this study added to our knowledge.

1. MIMO aims to provide a multilingual tool focusing specifically on health informatics and technologies.
2. The implications for medical informatics and digital health in the academic and industrial landscapes are essential to develop and to improve scientific, technical, educational, and industrial partnerships in medical informatics and digital health.
3. MIMO can be a pillar component of an environment supporting health sciences information professionals’ different activities, facilitating mutual understanding.

## CRediT authorship contribution statement

**Arriel Benis:** Conceptualization, Methodology, Validation, Formal analysis, Investigation, Resources, Writing – original draft, Writing – review & editing, Supervision, Project administration, Funding acquisition. **Julien Grosjean:** Software, Formal analysis, Investigation, Data curation, Writing – review & editing, Visualization. **Kévin Billey:** Software, Formal analysis, Investigation, Data curation, Writing – review & editing, Visualization. **Gustavo Montanha:** Validation, Investigation, Writing – review & editing. **Verena Dornauer:** Validation, Investigation, Writing – review & editing. **Mihaela Crișan-Vida:** Conceptualization, Validation, Investigation, Writing – review & editing, Funding acquisition. **Werner O Hackl:** Validation, Investigation, Writing – review & editing. **Lăcrămioara Stoicu-Tivadar:** Conceptualization, Validation, Investigation, Writing – review & editing, Project administration, Funding acquisition. **Stéfan J. Darmoni:** Conceptualization, Methodology, Software, Validation, Formal analysis, Investigation, Resources, Writing – original draft, Writing – review & editing, Visualization, Supervision, Project administration.

## Declaration of Competing Interest

The authors declare that they have no known competing financial interests or personal relationships that could have appeared to influence the work reported in this paper.
